# Outcomes of Dorsal and Ventral Buccal Graft Urethroplasty at a Tertiary Hospital in Uganda

**DOI:** 10.1155/2014/316819

**Published:** 2014-04-17

**Authors:** S. Kaggwa, M. Galukande, H. Dabanja, H. Luweesi

**Affiliations:** ^1^Department of Surgery, College of Health Sciences, Makerere University, Kampala, Uganda; ^2^Department of Surgery, Mulago National Referral Hospital, Kampala, Uganda

## Abstract

*Purpose*. Although the use of buccal mucosa in substitution urethroplasty has been practiced for some years, it has not been free of controversy over which surgical technique is the most appropriate to use. There is paucity of data in Sub-Saharan Africa about its success; this study presents the outcomes of dorsal and ventral buccal graft urethroplasty at a sub-Saharan tertiary hospital. *Methods*. This is a prospective study in which buccal mucosa was used for ventral and dorsal grafts; followup was up to two years. All patients provided informed written consent for the procedures. *Results*. Seventy-two patients with bulbar urethral strictures underwent buccal graft one-stage urethroplasty. Mean age was 55 years; etiology of the strictures was postinflammatory due to urethritis from sexually transmitted infections 97% (70/72) and trauma 3% (2/72). Buccal mucosa grafts were harvested from the cheek using a two-team approach. Grafts were placed on the ventral and dorsal urethral surfaces in 32 and 40 cases, respectively; the success rate was 84 and 80%, respectively. Repeated urethroplasty was successfully done among 10% (7/72) and patients reported resolution of symptoms in the follow-up period. *Conclusion*. There was no difference between dorsal and ventral onlay buccal graft outcomes for bulbar urethral strictures. The success rate was 80 to 84%.

## 1. Introduction

Urethral stricture is a chronic and common urological problem and difficult to manage fraught with high patient morbidity and stricture recurrence [1, 2].

Since the resurgence in the use of buccal mucosa grafts (BMG) in substitution urethroplasty in the late 1980s, there has been controversy over which surgical technique is the most appropriate for its application. In experienced hands the results of the ventral and dorsal onlay of BMG for bulbar urethroplasty are the same [2].

BMG has become an ideal urethral substitute because of ease of harvest, surgical handling characteristics, hairlessness, and compatibility in a wet environment and it is early in growth and graft survival; because of these unique characteristics, buccal mucosa has gained popularity in the realm of reconstructive urology.

Standard bulbar urethroplasties using BMG should have a lifetime success rate approaching 92% [3, 4].

There is paucity of data on this subject in Sub-Saharan Africa; the purpose of this study was to compare ventral and dorsal onlays for bulbar urethroplasty in a low resource setting.

## 2. Methods

### 2.1. Design

This was a prospective study.

### 2.2. Settings

It is performed at a tertiary referral hospital at Mulago over a two-year duration (2010–2012).

### 2.3. Recruitment

Consecutive patients presenting with difficulty in passing urine (weak stream, incomplete emptying, and frequency) due to a penile urethra stricture were recruited in two phases: over a 12-month period for dorsal onlay grafting and the subsequent 12 months another set was recruited for ventral onlay grafting.

Those with concomitant symptomatic prostate enlargement were excluded.

### 2.4. Ethical Considerations

All patients provided informed written consent for the procedures.

### 2.5. Data Collection and Analysis

We collected data using a questionnaire; data were extracted from the patients files and entered in an excel spread sheet and then exported to SPSS version 17 and analysis was done using descriptive statistics.

### 2.6. Study Variables

A urethral stricture was diagnosed through history taking and physical examination and confirmed on performing a cystourethrogram (ascending and micturating urethrogram). Recurrence (failure) was defined as a need for additional surgical procedure or filiform bougienage within the follow-up period. The followup was up to 2 years.

Variables studies included level of client satisfaction; patients were asked if they were satisfied or not with the ease of voiding urine, erectile dysfunction (ED), and postvoid dribbing. ED was considered if it was of sudden onset and did not exist prior to surgery. The question asked was if at any time after surgery erections were insufficient for intercourse in comparison to before surgery [5]. Postvoid dribbling was defined as a dribble or urine leak a few minutes after voiding.

The success of urethroplasty was from the patient self-reports of improved urine stream, complete emptying, and reduced frequency.

### 2.7. Surgical Techniques

We used a two-team approach for graft harvest and perineal dissection. The perineal dissection team laid the stricture open to determine its length before the graft harvest team proceeded.

#### 2.7.1. Buccal Harvest

The buccal harvest technique below was described before in 2011 [2].

Patients were instructed to use a mouthwash containing chlorhexidine in the preoperative period. All the patients received intraoperative antibiotics (Ceftriaxone and Metronidazole) intravenously before the oral mucosa was excised. The face and cheek were prepped with 0.5% chlorhexidine and draped in the usual sterile manner with sterile linen. Three 3/0 silk sutures are placed through the lip to provide traction. A mouth gag and a retractor were used to facilitate exposure. Using a marking pen, the graft was outlined 2.5 cm wide and as long as required.

0.5% of bupivacaine with epinephrine were injected underneath the graft for preemptive analgesia and intraoperative haemostasis. The graft was incised and dissected off of the buccinators muscle, while avoiding Stensen's duct. The graft was pinned out and thinned on the back table. It was kept in saline until the time of transfer. The donor site was left unstitched as a routine practice [6–8].

Donor site is packed with a gauze piece soaked in adrenaline and lignocaine. The oral pack is removed in the evening and the patient is asked to rinse his mouth with cold water and dilute mouthwash. The cavity was inspected for any bleeding and the patient asked to start cold oral liquids in the evening. In a day or two the patient was advised to shift to semisolid, nonspicy diet and can consume normal diet as soon as he can tolerate it.

#### 2.7.2. Surgical Technique: Ventral Urethroplasty [2]

Patient was placed a high lithotomy position, properly padded and secured. Subsequently the patient's perineum was prepped with 0.5% of chlorhexidine and draped in normal sterile fashion.

A 22F catheter was used to delineate the urethral contour and to determine the exact location of the distal portion of structure; a number 15 blade scalpel was used to incise the urethra over the urethral catheter and the urethral stricture is opened completely. The lumen of the structure was intubated with either an 8F-feeding tube or with a small (<8 mm) guide wire, and the stricture was incised until normal urethra was identified. Both proximal and distal urethral stumps were bougied to ensure they are wide open to 30F.

The buccal mucosa was then sewn onto the ventral defect using a running and interlocking 5/0 vicryl suture, for a good seal. An F16 silicone catheter was placed through the urethra. The tunic of the spongiosum is closed over the graft for a well vascularized bed. The bulbospongiosus muscle was closed with a running 3/0 vicryl and the skin was closed with multiple vertical mattress stitches using 2/0 vicryl rapide. Prior to skin closure, the wound is anaesthetized with 0.5% bupivacaine.

#### 2.7.3. Dorsal Urethroplasty

The technique employed was similar to the one outlined by Barbagli et al, 2006 [9].

For the follow-up period, the catheters were removed on the 21st day after the operation was done. No uroflowmetry was done. It was not available to clients. And no pericatheteric urethrograms were done.

## 3. Results

Table 1 shows the demographic, clinical characteristics and the outcomes of the buccal graft procedures.

The mean age was 55 years, the age range was 10–86 years, and the recurrence rates were 16–20%.

40 dorsal grafts provided success in 32 (8 failed cases) and 32 ventral grafts provided success in 27 (5 failed cases).

### 3.1. Etiology

70 were postinflammatory (infection). 2 were trauma related.

## 4. Discussion

We found that dorsal and ventral onlay grafts offer the same performance; the recurrence rates were 16–20%, comparable with data from elsewhere [1, 2, 9–13]. The procedures were fairly easy to perform; the mean operating time was 2 hours. The average length of hospital stay was 5 days. Client satisfaction was high; the number of complaints was minimal.

The etiology of these strictures was postinflammatory (infection) for the most part (97%) as is common in low resource settings where urethritis due to sexually transmitted diseases is prevalent [1].

For the most part, the outcome for both dorsal onlay grafts and ventral onlay grafts in bulbar urethroplasty is similar. The findings in this study affirm that the ventral onlay technique is, however, less cumbersome and therefore more suitable for surgeons new to the practice of urethroplasty. The complications associated with ventral onlay techniques can be minimized by meticulous surgical technique, but in series with longer followup, complications still tend to be more prevalent. In urethroplasty, two-stage dorsal onlay of BMG (after complete excision of the scarred urethra) still provides the best results, although in certain circumstances a one-stage dorsal onlay procedure is possible [10]. In general, tube graft procedures in the management of bulbar urethral strictures are associated with much higher rates of recurrence and should therefore be avoided.

The most common graft materials in use today are buccal mucosa (BM), preputial skin (when available), and penile and preputial skin flaps with their own blood supply. The most appropriate use of these materials has long been the subject of controversy, especially in terms of which type of tissue and whether as a graft or flap and at which site along the urethra [8–13].

The use of BM in urethral surgery was first described in 1941 [14] but not reported again until the late 1980s. Since then, it has proved to be a versatile graft material well suited to repair the urethra [10, 15–17] because it is a wet epithelium, which is easily harvested and amenable to surgical manipulation, has a privileged immunity rendering it less prone to infection, and is more resistant to stricture recurrence than skin particularly in the presence of lichen sclerosus, previously known as balanitis xerotica obliterans. In this study, we experienced 10% (7 patients) stricture recurrence and the three had perineal wound infections. BM also has a dense submucosa with a dense capillary network that facilitates the early imbibition of nutrients from the wound bed as well as early inosculation of neovasculature [10, 15–18]. The graft is harvested either from the inner aspect of one or both cheeks, from the posterior lower lip, or in cases where extensive substitution is necessary, from all three sites. Several papers have looked at the morbidity associated with harvesting the BM graft, and all conclude that morbidity is lower with inner cheek harvest than lower lip, because these patients tend to have a lesser degree of discomfort and a lower rate of paraesthesia (secondary to mental or lingual nerve injury) postoperatively [19, 20]. We however technically preferred the lower lip approach and had no adverse events.

When substitution procedures are necessary, historically various tissues have been used including genital (penile and scrotal) skin, extragenital skin, bladder mucosa, and buccal mucosa. These tissues have been used as either pedicled flaps with their own blood supply or as free tissue grafts. The use of colonic mucosa has also been reported, but this is not validated yet and seems to involve a significantly greater degree of morbidity for graft harvest than any of the other methods mentioned.

## 5. Conclusion

There was no difference between dorsal and ventral onlay buccal graft outcomes for bulbar urethral strictures. The success rate was 80 to 84% after an average follow up period of one year.

## Limitations

This was a single-site study and by one operator experienced in dealing with urethral strictures. CUG (cystourethrogram) was only done for those experiencing difficulties in passing urine; no follow-up cystoscopies were done (for any patient). Poor oral hygiene and the use of tobacco were not factored in as potential confounders; these factors are known to influence the outcome of the BMG [21]. The mean follow-up period of one year was short to draw definite conclusions, though a general sense is given for ease of performing the procedure and short term outcomes especially of urethral patency.

## Figures and Tables

**Table 1:** Clinical characteristics and study outcomes of buccal graft urethroplasty.

Variables	Dorsal *n* = 40	Ventral n = 32
Age (years)		
Mean	55	54
Range	12–84	10–86
Mean operating time	2 hours	2 hours
Average length of hospital stay	5 days	5 days
Stricture length (cm)		
Mean	4.5	4.0
Stricture site	Bulbar	Bulbar^††^
Outcomes		
Immediate passage of urine	40	32
Recurrence of stricture	8	5
Follow-up events		
Filiform bougienage	4	2
Repeated urethroplasty*	4	3
Urethrocutaneous fistula	Nil	Nil
**Erectile dysfunction	Nil	Nil
Postvoid residual urine volume	Not taken	Not taken
Urinary flow rate	Not done	Not done
Donor site complication—Numbness, tightness (Buccal site)	Nil	Nil
Perineal infection	1	2
Characteristics of failures		
Mean age	59	64^†^
Range	30–80	32–70

*With good outcomes (patients passing urine normally during the follow-up period).

**ED questions were posed to those for whom it was appropriate (i.e., had a sexual partner).

^†^One outlier excluded a 10-year-old with a midpenile stricture.

^††^With the exception of one pendulous urethral stricture.

## Abbreviations

BMG: Buccal mucosa grafts
BM: Buccal mucosa
CUG: Cystourethrogram.
